# Geographic variations of the bird-borne structural risk of West Nile virus circulation in Europe

**DOI:** 10.1371/journal.pone.0185962

**Published:** 2017-10-12

**Authors:** Benoit Durand, Annelise Tran, Gilles Balança, Véronique Chevalier

**Affiliations:** 1 University Paris Est, Anses, Laboratory for Animal Health, Epidemiology Unit, Maisons-Alfort, France; 2 Cirad, UMR TETIS, Montpellier, France; 3 Cirad, UMR ASTRE, Montpellier, France; 4 Institut Pasteur du Cambodge, Epidemiology and Public Health Unit, Phnom Penh, Cambodia; National Institute of Health, ITALY

## Abstract

The structural risk of West Nile Disease results from the usual functioning of the socio-ecological system, which may favour the introduction of the pathogen, its circulation and the occurrence of disease cases. Its geographic variations result from the local interactions between three components: (i) reservoir hosts, (ii) vectors, both characterized by their diversity, abundance and competence, (iii) and the socio-economic context that impacts the exposure of human to infectious bites. We developed a model of bird-borne structural risk of West Nile Virus (WNV) circulation in Europe, and analysed the association between the geographic variations of this risk and the occurrence of WND human cases between 2002 and 2014. A meta-analysis of WNV serosurveys conducted in wild bird populations was performed to elaborate a model of WNV seropositivity in European bird species, considered a proxy for bird exposure to WNV. Several eco-ethological traits of bird species were linked to seropositivity and the statistical model adequately fitted species-specific seropositivity data (area under the ROC curve: 0.85). Combined with species distribution maps, this model allowed deriving geographic variations of the bird-borne structural risk of WNV circulation. The association between this risk, and the occurrence of WND human cases across the European Union was assessed. Geographic risk variations of bird-borne structural risk allowed predicting WND case occurrence in administrative districts of the EU with a sensitivity of 86% (95% CI: 0.79–0.92), and a specificity of 68% (95% CI: 0.66–0.71). Disentangling structural and conjectural health risks is important for public health managers as risk mitigation procedures differ according to risk type. The results obtained show promise for the prevention of WND in Europe. Combined with analyses of vector-borne structural risk, they should allow designing efficient and targeted prevention measures.

## Introduction

West Nile disease (WND) is caused by the West Nile virus (WNV) (*Flavivirus*, *Flaviviridae*). The transmission cycle involves wild and domestic birds as main hosts and mosquitoes, mainly of the *Culex* genus, as vectors. Under favourable environmental conditions, this cycle may be amplified and lead to human and horse infections. The latter two are considered dead-end hosts [1]. Most human cases remain asymptomatic. However, around 30% of infected people get sick, with symptoms ranging from a flu syndrome to encephalitic diseases, with recent reported fatality rates ranging from 3 to 17% [2,3]. Ten percent of horses infected by WNV present neurological disorders [4,5]. WNV has been circulating in the Mediterranean Basin at least since the 1950s [6]. Most of human and/or equine cases were caused by strains belonging to lineage 1a, characterized by a moderate pathogenicity for horses and humans and a limited or no pathogenicity for birds [7]. However, since 2000, WNV epidemiological pattern has evolved with an increase of mortality in some bird species, and a higher incidence of animal and human neurological cases. Furthermore, lineage 2 strains, so far confined to the south of the Sahara, has been first detected in 2004 in Hungary and thereafter in several countries of central and southern Europe [8–13].

The structural risk of WND results from the usual functioning of the socio-ecological system, which may more or less favor the introduction of the pathogen, its circulation and the occurrence of clinical cases. The conjectural risk corresponds to the combination of external features, such as climatic events, that may modify the assembly of avian and mosquito species in their composition and/or their competence, and favor or not virus transmission. Geographic variations of structural risk of WND emerge from the local interactions between three components: (i) reservoir hosts, (ii) vectors, both characterized by their diversity, abundance and competence; and the (iii) socio-economic context that impacts the exposure of human to infectious bites [14–17]. Local European bird population composition and abundance strongly vary in space and time. The intrinsic receptivity (i.e. the permissiveness to infection and the capacity to replicate and transmit the virus to another host) [18] of European bird species to WNV infection has been poorly studied. Their exposure to mosquito bites varies according to their physical characteristics (size and weight), as well as to their eco-ethological features [18–22]. According to life history theory, “fast-lived” species would invest minimally in adaptive immunity, contrary to “slow-lived” species. As a consequence, (i) the potential to transmit a given pathogen would be higher for “fast-lived” species than for “slow-lived” ones [23]; (ii) because of the life-long WNV immunity, a constant exposure to the same force of infection will lead to a higher proportion of susceptible individuals in fast-lived species than in “slow-lived” ones. Fast-lived species are thus assumed to play the main role in WNV circulation. Because of their short lifespan (a few years) and assuming that mortality due to infection is negligible, species-specific seroprevalence in these birds can be considered a relevant indicator for the species implication in WNV epidemiological cycle. Majority of fast-lived birds are passerines. A passerine is any birds of the order) Passeriformes, the largest and most diverse commonly recognized clade of birds. The breeding behaviour of these birds is diverse: most species are solitary nesters, but other may be colonial nesters, defending only the nest site and a small area immediately adjacent to it. Some species build individual nests close together in a colony. Nest sites are varied: they include holes in the ground, trees, banks, and rock crevices; they may be on ledges, on the surface of the ground, within the larger nests of other species or near wasp nests and in a wide variety of vegetation—grasses, shrubs, and trees.

The objective of this study was to analyse the geographic variations of the bird-borne structural risk of WND in Europe, and analyse the association between this structural risk and the occurrence of WND cases across the European Union (EU)

## Materials and methods

A meta-analysis of WNV serosurveys conducted in wild bird populations was first performed to elaborate a statistical model of WNV seropositivity in European fast-lived bird species, according to several eco-ethological traits likely impacting on bird exposure to mosquito bites. The statistical model was then used to rank 150 European fast-lived bird species according to the predicted seropositivity level. These results were combined with the distribution in Europe of these 150 bird species to derive geographic variations of the bird-borne structural risk of WNV circulation. The association between this structural risk, considered as a predictor, and the occurrence of WND cases across the European Union (EU) was finally analysed.

### Bird ecological traits

Several ecological traits were considered as potential risk factors for WNV seropositivity. Bird migration induces a greater exposure to WNV in birds that spent part of the year in sub-saharian endemic areas [20]. Body mass is linked to WNV seropositivity by two mechanisms: (i) larger birds release more CO_2_ and are thus more attractive for mosquitoes, (ii) body mass is linked to life span and the duration of WNV antibodies is lifelong: for a given exposure level to WNV, older birds (i.e. birds of larger species) will be more often seropositive than younger birds (i.e. birds of smaller species). The breeding season may impact the exposure of birds to infectious bites, (e.g. male specimens of territorial species), especially during nesting. Adult birds are less mobile during the breeding season period than during the rest of the year. The nest shape and location modulate the exposure of adults and chicks to mosquito bites, as does the feather coverage for chicks [24]. Many mosquito’ species exhibit vertical height specialization for host seeking [25]: birds building nests on the ground may beless exposed than birds building medium nests. Because of a lower avian biodiversity and a different vector species composition, the use of urban habitats may impact bird exposure to infectious bites. Finally, the gregariousness may impact exposure to infectious bites, either towards an increase because large groups of birds are more attractive for vectors than solitary individuals, or towards a decrease: solitary individuals may be more frequently bitten than individuals of a large group because of a higher mosquito-to-bird ratio [26].

Since most passerine species fall within the range of about 15 to 50 grams in weight, a dataset was generated for 150 European bird species weighting <50g in adult specimens (see S1 Table) [27]. In this database, each species was associated with the adult body mass (in grams), the nest height (3 classes: nest built on the ground, <4 meters, >4 meters: birds nesting above 4 meters, usually nest in bush, building holes or bird-houses, and most of them weight less than 50g), the exposure of nestling (altricial species–with no or minimal feather coverage, *vs* precocial species), the use of urban/suburban habitats (absence or rare *vs* other cases), the nocturnal gregariousness (birds spend the night in large groups, yes/no), the breeding sociality (birds congregate during the breeding period, yes/no) and the migratory status (migratory if most of the birds spent the European winter in sub-saharian regions, non-migratory otherwise). Ecological traits were defined and assigned to each species according to [27].

### Geographic distribution of bird species in Europe

The distribution area of each of the 150 European bird species weighing <50g was obtained from BirdLife International (BirdLife International and NatureServe, 2012, “Bird species distribution maps of the world”, available from http://www.birdlife.org/). Provided maps give, for each species, the contours of the European zones where it can usually be found. However, inside these zones, the presence of birds may strongly vary locally, according to the biotopes. The geographic distribution of birds was thus refined, taking into account the habitat preferences of each bird species provided in Cramp, et al [27]. To do so, a global land cover map produced by the European Space Agency (Source Data: Globcover ESA/ESA Globcover Project, led by MEDIAS-France/POSTEL) was used to define distinct habitats. For each of the 25 land cover classes, a binary variable (presence/absence) was associated to each bird species (see S2 Table). For each bird species, this dataset was combined with the Globcover map and the repartition area (used as a mask) to generate a map of the geographic distribution of the species, with binary 100x100 m pixels set to 0 outside the repartition area, or inside the repartition area for non-favourable habitats, and pixels set to 1 inside the repartition area for favourable habitats.

### WNV seroprevalence data in wild birds

To build a statistical model linking WNV seropositivity in European fast-lived bird species, and eco-ethological traits, a systematic review of WNV serosurveys conducted in European wild bird populations was performed. This review was done according to the methodology provided in Moher et al [28] (see S3 Table). PubMed and Scopus databases were searched to identify relevant serological studies published after the 1^st^ January of 2000, using the following query: (West Nile [title]) AND (bird* [title/abstract] OR sero* [title/abstract]) AND ("2000/01/01" [Date—Publication]: "3000" [Date—Publication]). Inclusion criteria used to identify the final list of publications were the geographic area where the survey was performed (Europe and Maghreb where many European birds spent part of their life), and the report of original serological data in birds. Reviews, experimental studies, descriptions of clinical disease, pathogenicity and diagnosis, transversal and longitudinal surveys conducted in domestic or captive birds were discarded, as well as studies based on dead wild birds only. The resulting articles were read, and the relevant papers cited by these articles but not identified by the above selection procedure were then included in the review. Studies for which the serological results were not given at the species level and serological results obtained using laboratory methods other than virus neutralization tests (VNT) were discarded. The datasets of serological results selected were collected in a database, with 4 variables: the study id, the species name, the number of tested birds and the number of positive birds (see S4 Table).

### Statistical modelling of WNV seropositivity in wild birds

WNV seroprevalence data were analysed using logistic mixed models. The dependent variable was the serological status of birds. The study was treated as a random effect, to account for differences between studies according to the country, year and month where the birds were caught and sampled, as well as to differences in study designs such as the cut-off points used in VNT. Two models were compared based on the Akaike Information Criterion (AIC) [29]. In the “traits-based model”, the above potential risk factors for WNV seropositivity were included as fixed effects, whereas in the “species-based model” the single fixed effect was the species. The absence of significant multicollinearity was checked for the traits-based model by verifying that the variance inflation factor was <5 for each of the fixed effects. Parametric bootstrap (1000 repetitions) was used to compute the confidence intervals of the odds-ratios. The fit of the model was evaluated using the receiver operating characteristic (ROC curve) and by computing the area under the curve (AUC). A split sample cross-validation was conducted to evaluate the reliability of the traits-based model (2/3 of the dataset randomly selected for estimation, and 1/3 for prediction, 100 repetitions). The mean AUC was compared with that obtained using the full dataset: the reliability of the model was judged satisfactory if the difference was low (less than 0.1).

### Geographic variations of bird-borne structural risk of WNV circulation

The traits-based model was used to predict the seroprevalence in the 150 European bird species weighing <50g. Only fixed effects were taken into account. For each species, a point estimate was produced using the original dataset, and the species rank was calculated based on these predictions. Parametric bootstrap (100 repetitions) was used to compute the corresponding confidence intervals. The corresponding 100 seroprevalence predictions were then used to build 100 bootstrapped risk maps, by computing the value of each pixel as follows:

$$R_{k}(i,j)=\sum_{sp=1}^{150}\left[H(sp,i,j)\times P_{k}(sp)\right],\;\mathrm{where}:$$

- *R*_*k*_(*i*,*j*) is the risk value associated to pixel of coordinates (*i*, *j*), based on the predictions of the *k*th bootstrap repetition,
- *H*(*sp*,*i*,*j*) is 1 if the pixel of (*i*, *j*) is located in the repartition area of species *sp* and if the habitat found in this pixel is favourable to this species; and 0 otherwise,
- *P*_*k*_(*sp*) is the predicted seroprevalence for species *sp*, according to the *k*th bootstrap repetition.

The 100 bootstrapped risk maps obtained were finally summarized into a single risk map associating to each pixel a 0–100 value: the proportion of the 100 bootstrapped risk maps in which this pixel was above the 90^th^ percentile of the distribution of pixel values. Considering as “high-risk” pixels for which the risk was above the 90^th^ percentile of the distribution, this final risk map thus indicated, for each pixel, the probability to be a high-risk pixel.

### Comparison of bird-borne structural risk of WNV circulation with epidemiological data

The relationship between the final risk map and the occurrence of WND cases in human was analysed using a logistic model. Epidemiological data were the number of WND cases per administrative district (NUTS3 level) between 2002 and 2014 in the European Union. The sensitivity of case detection was assumed similar between administrative districts and countries. This dataset was generated from several sources, as described in [30]. Two quantitative predictors were analysed: the proportion of pixels for which the predicted risk was >50% (i.e. pixels with a >50% probability to be high-risk pixels), and the proportion of pixels for which the predicted risk was >80% (i.e. pixels with a >80% probability to be high-risk pixels). The district human population size was added as an adjustment variable, and treated as qualitative ordinal variable with 4 classes (quartiles of the distribution). The absence of significant multi-collinearity was checked as described above. The odds-ratios associated to the predictors were computed, and the fit quality of the model was evaluated by computing the AUC of the ROC curve. The reliability of the model was evaluated using a split-sample cross-validation (90% of the dataset for estimation and 10% for prediction, 10 repetitions). The predicted district-level risk of WND cases was computed and mapped using a choropleth map.

## Results

**Fig 1:**
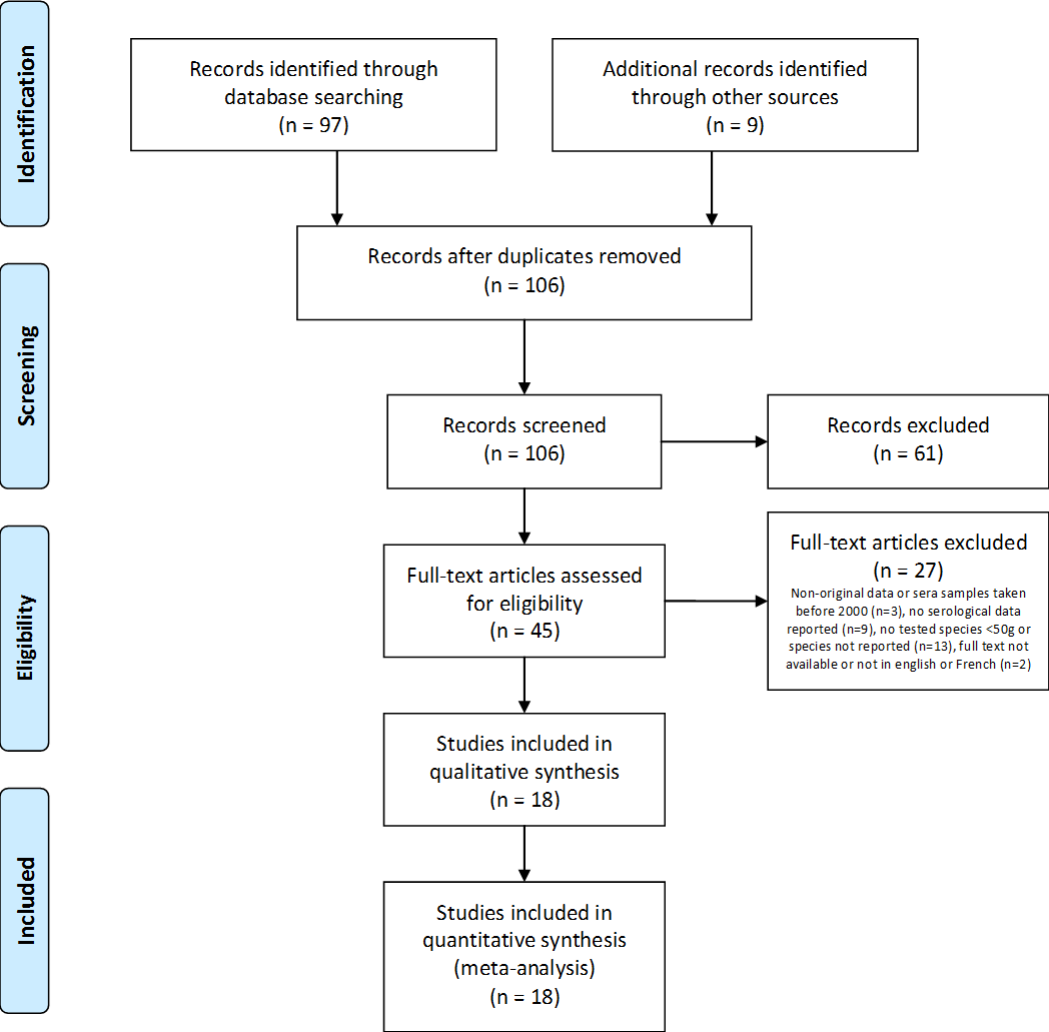
PRISMA flow diagram for included paper selection (adapted from [28]).

The PubMed and Scopus initial query (launched on 2016-01-20) returned 276 articles, and six articles identified through other sources were also considered. The results of the PRISMA method for paper selection are provided in Fig 1. Application of inclusion/exclusion criteria resulted in 18 papers (Table 1) describing studies conducted in 10 countries -9 European countries and Morocco, and providing original data of WNV seroprevalence in wild birds of species weighing <50g in adult specimens. These studies reported a total number of 7685 serological results in 64 bird species (21 families), of which a total number of 103 birds of 32 species and 15 families were seropositive.

**Table 1:** WNV seroprevalence data in wild birds of Europe and Maghreb, reported in scientific publications between 2000 and 2015.

**Country**	**Year**	**All bird species**		**<50g bird species**		**Ref.**
		Tested birds (species)	Positive birds (species)	Tested birds (species)	Positive birds (species)	
**France**	2000	460 (5)	13 (3)	117 (1)	0 (0)	[31]
	2004	432 (32)	19 (8)	370 (15)	18 (7)	[32]
	2004	227 (3)	4 (2)	196 (2)	1 (1)	[33]
	2005–2007	2350 (13)	11 (3)	2848 (7)	7 (1)	[34]
**Spain**	2003–2005	1213 (72)	126 (24)	462 (31)	10 (5)	[20]
	2004	524 (25)	22 (4)	472 (18)	18 (3)	[22]
	2013	149 (32)	1 (1)	121 (25)	1 (1)	[35]
**Germany**	2000–2005	3399 (87)	53 (5)	169 (6)	8 (5)	[36]
	2005–2009	1086 (57)	41 (10)	7 (5)	2 (1)	[37]
	2011–2013	902 (88)	45 (20)	21 (2)	4 (2)	[38]
**Italy**	2006–2008	1405 (47)	3 (3)	1175 (33)	2 (2)	[39]
	2012–2013	233 (43)	16 (6)	11 (5)	1 (1)	[40]
**Czech rep.**	2004–2006	391 (28)	23 (10)	311 (16)	16 (9)	[41]
**Sweden**	2005–2006	1935 (104)	2 (2)	726 (7)	2 (2)	[42]
**Romania**	2007–2008	713 (20)	37 (9)	327 (6)	8 (2)	[43]
**Poland**	2010–2014	474 (15)	63 (2)	20 (2)	1 (1)	[44]
**Serbia**	2012	133 (45)	7 (3)	33 (9)	0 (0)	[45]
**Morocco**	2008	346 (16)	12 (3)	299 (9)	4 (1)	[46]
**Total**				**7685 (64)**	**103 (32)**	

The AIC was 359 for the traits-based model and 369 for the species-based model: the traits-based model fitted seroprevalence data more parsimoniously than the species-based model. Several traits were associated with WNV seropositivity (Table 2). The body mass was positively linked to WNV seropositivity. A protective effect of nest location was observed. Species that build their nest on the ground appeared more exposed to WNV (OR: 2.7) than species that build their nest at an intermediate height. Seropositivity was significantly higher in altricial species than in precocial species (OR: 2.1). There was no statistical link between the use of urban/suburban habitats, the nocturnal gregariousness, the breeding sociality and the migratory status andWNV seropositivity. The AUC of the ROC curve was 0.85 (95% confidence interval [CI]: 0.81–0.89), and the split-sample cross-validation resulted in a mean AUC of 0.80, suggesting a reasonably good reliability of the traits-based model. This model was used to predict the seroprevalence in the 150 European bird species weighing <50g.

**Table 2:** Model of WNV seropositivity in birds weighing less than 50 grams, based on species ecological traits.

**Ecological trait**	**Odds-ratio (95% confidence interval**^a^**)**	**p-value**
**Intercept**	0.007 (0.001–0.02)	<0.001
**Body mass^b^:**		
** <12 grams**	Reference	
** 12–18 grams**	2.3 (1.0–5.4)	0.02
** 18–28 grams**	3.6 (1.5–8.9)	0.004
** >28 grams**	3.1 (1.4–8.1)	0.006
**Nest height:**		
** Above ground and <4 meters**	Reference	
** On the ground**	2.7 (1.4–4.9)	0.003
** >4 meters**	0.4 (0.1–1.2)	NS^c^
**Exposure of nestlings:**		
** Precocial species**	Reference	
** Altricial species**	2.1 (1.2–3.8)	0.01
**Use of urban/suburban habitats:**		
** Absence or rare**	Reference	
** Other cases**	1.0 (0.4–2.2)	NS
**Nocturnal gregariousness:**		
** No**	Reference	
** Yes**	0.8 (0.5–1.5)	NS
**Breeding sociality:**		
** No**	Reference	
** Yes**	0.6 (0.2–1.9)	NS
**Migratory status:**		
** No**	Reference	
** Yes^d^**	0.7 (0.4–1.7)	NS

^a^Parametric bootstrap confidence intervals.

^b^Classes based on quartiles of body mass in the 150 European species weighing less than 50 grams.

^c^Not significant.

^d^Most of the birds spent the European winter south to the Sahara.

**Fig 2:**
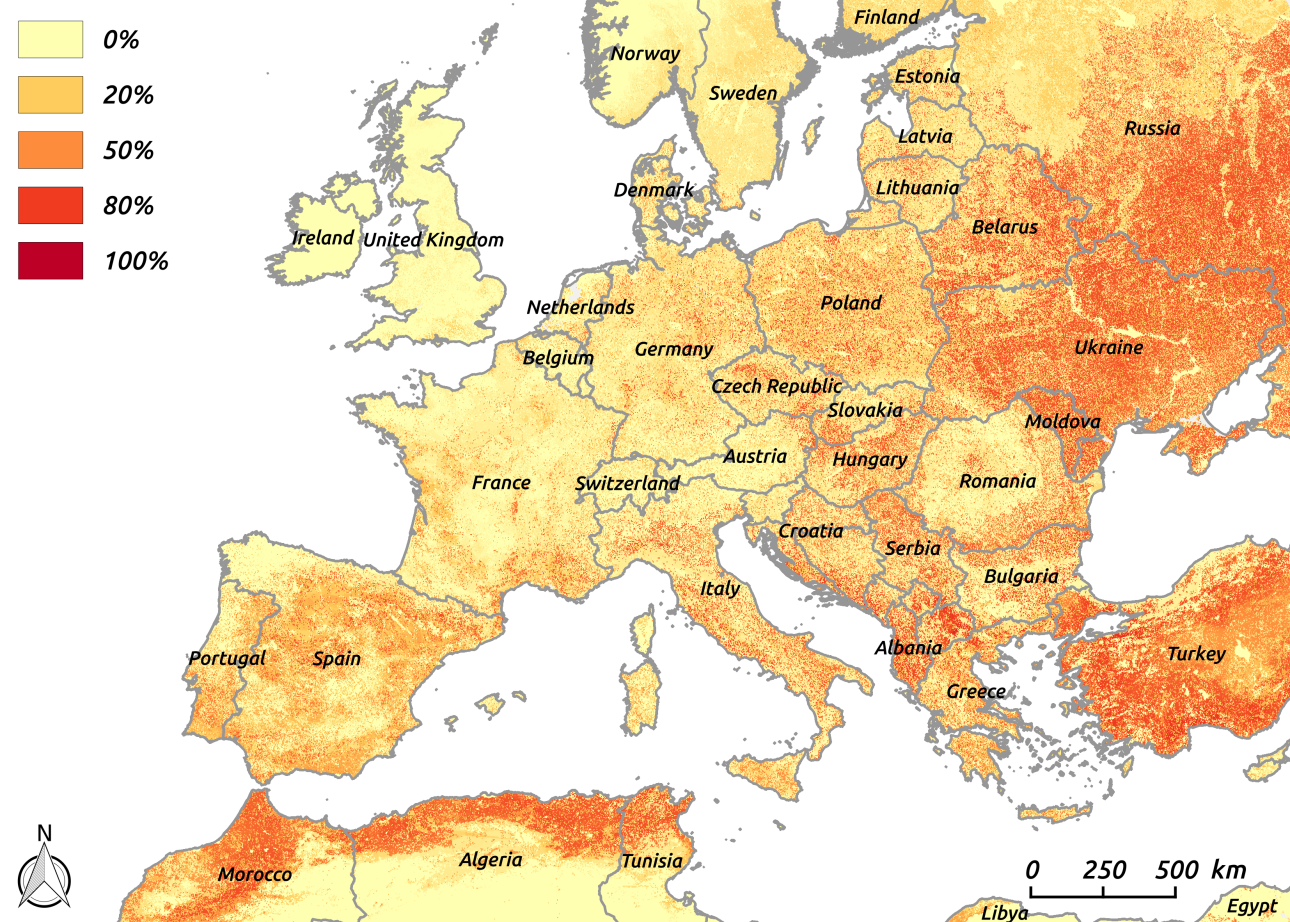
Map of the predicted structural bird-borne risk of WNV circulation in Europe: Geographic variations of the probability of high-risk areas (pixel-specific probability of belonging to the 10% pixels with the highest predicted structural bird-borne risk).

The bird-borne structural risk map showed areas with a strong probability (>80%) to be high-risk in Maghreb countries (Morocco, Algeria and Tunisia), Turkey, Ukraine, Moldavia and Western Russia. Within The European Union, high-risk areas were mainly located in Mediterranean countries (Fig 2).

Between 2002 and 2014, human cases of WND were reported in 105 of 1311 administrative districts (NUTS3 level) of the European Union (http://ecdc.europa.eu/en/healthtopics/west_nile_fever/West-Nile-fever-maps/Pages/historical-data.aspx). The logistic model showed a strong association between the occurrence of WND cases in a given district and the presence of high-risk pixels in that district (Table 3) (OR of 7.4 for a 5% increase of the density of pixels having >80% probability to be high-risk, OR of 1.7 for a 5% increase of the density of pixels having a >50% probability to be high-risk). The AUC was 0.84 (95% CI: 0.80–0.89), and the split-sample cross-validation resulted in a mean AUC of 0.83 (range: 0.65–0.94). The ROC curve was used to compute the probability threshold that maximized the sum of the sensitivity and of the specificity of the model prediction (also known as the Youden’s J statistic): 0.05. Using this threshold, the sensitivity of the model, considered as a predictor of the occurrence of WND cases, was 86% (90 of 105 districts with reported WND cases were correctly classified, bootstrap 95% CI: 0.79–0.92). The specificity was 68% (824 of 1206 districts without reported WND cases were correctly classified, bootstrap 95% CI: 0.66–0.71).

**Table 3:** Model of occurrence of WND cases in humans in administrative districts of the European Union between 2002 and 2014, according to the predicted structural bird-borne risk of WNV circulation and to the population size.

**Variable**	**Odds-ratio (95% confidence interval)**	**p-value**
**Intercept**	0.008 (0.004–0.016)	<0.0001
**Proportion of pixels classified high-risk with a probability >0.5^a^**	1.7^b^ (1.5–1.9)	<0.0001
**Proportion of pixels classified high-risk with a probability >0.8 ^a^**	7.4^b^ (2.9–24.0)	0.0001
**Population size (x10^5^)**		
** <1.3**	Ref.	
** 1.3–2.5**	1.5 (0.7–3.4)	0.34
** 2.5–4.8**	3.5 (1.8–7.5)	0.0006
** >4.8**	3.1 (1.5–6.5)	0.002

^a^Pixels with an predicted risk above the 90^th^ percentile of the risk distribution in >50% (resp. 80%) of bootstrapped risk maps.

^b^Odds-ratios computed for an increase of 0.05 of the proportion.

**Fig 3:**
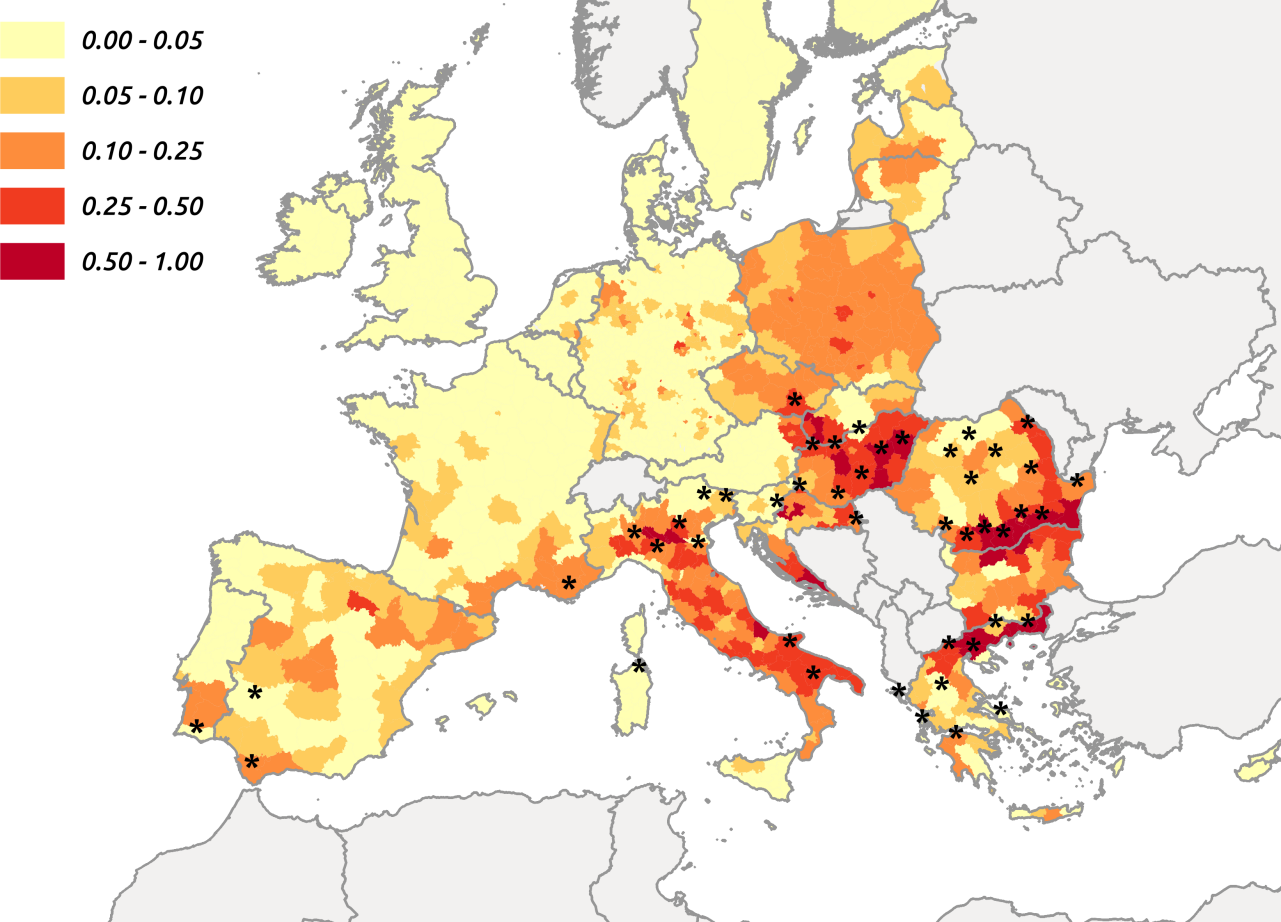
Map of the predicted occurrence of WND cases in administrative districts of the European Union between 2002 and 2014, according to the predicted structural bird-borne risk of WNV circulation and to the human population size (stars: Districts having reported WND cases).

Based on the logistic model, a choropleth map of the predicted probability of WND occurrence was generated. The bounds of the probability classes were based upon the above probability threshold, with the 1^st^ class (having the lightest colour) corresponding to predicted probabilities below the threshold (thus classified negative according to this threshold) (Fig 3). This map confirmed that most districts having reported WND cases between 2002 and 2014 were associated with medium to high predicted probabilities.

## Discussion

The risk of WNV transmission results from a combination of the structural risk, ie the usual functioning of the socio-ecological system, and the conjectural risk that corresponds to external features, such a climatic events. Published studies that aimed at producing WND risk maps for Europe [30,47] or North-America [17,48] mixed in a single model the determinants of the structural risk with those of the conjectural risk. However, the distinction between both types of risk (and the knowledge of their respective weights in WND overall risk) is important for public health risk managers. Indeed, the mitigation procedures differ according to the risk type: preventive measures, ranging from land use planning to vaccination campaigns targeted on areas at risk, are adapted to the structural risk, whereas the focus is rather on preparedness, early warning, emergency planning and disease control for the conjectural risk. This study aimed at analysing the geographic variations of the bird-borne structural risk of WND in Europe, and analyse the association between this structural risk and the occurrence of WND cases across the European Union (EU).

The capacity of a local bird population to support WNV circulation is governed by two parameters that operate on distinct levels [18]: the intrinsic receptivity of birds to WNV, that is based on genetic determinants, whereas the exposure of birds to mosquito bites is rather based on eco-ethological determinants. A previous study, conducted in North-American zoo animals and bird populations, showed a significant contribution of intrinsic receptivity to the variations of seroprevalence among bird species [18]. In our study, a species-based model of seropositivity risk (with a single explicative variable, the species, which represented both receptivity and exposure) was compared with a traits-based model (including several variables describing eco-ethological traits that represented exposure only). According to AIC, the traits-based model better fitted the dataset than the species-based model. This corroborates results of observational studies [49] and of experimental studies conducted on European passerines that did not show marked differences between species for viraemia, viral load in organs, mortality and immune response whatever the strain involved [50–54]. Therefore, and even if 16 out of 18 studies used for this analyses were performed after 2004 when lineage 2 was firstly isolated in Europe (9), our results suggest that for European short-lived bird species, the infection risk is mainly driven by bird exposure to mosquito bites, the receptivity contribution to this risk being not a major driver. However, the lineage, 1 or 2, may influence mosquito behavior and competency, thus serological status of exposed birds. This latter component should be integrated in the future.

Seven eco-ethological traits were used to model, at the species level, how physical characteristics and behaviour modulate bird exposure to mosquito bites. The positive relationship between body mass and seropositivity, already observed in a previous study [20], was confirmed. Two eco-ethological traits representing exposure during the breeding season were significantly associated with seropositivity: the exposure of nestlings and the nest location. The low feather coverage in altricial species was associated with a higher seropositivity risk, as already observed [18]. The link between nest shape and seropositivity risk had been observed in African resident birds [19]. Here a significantly higher risk in species that build their nest on the ground was observed, which may be attributed to a better host availability for mosquitoes. The encounter-dilution hypothesis predicts that the per-capita number of mosquito bites should decrease within larger groups. Verified in North-American bird populations [55,56], this hypothesis was not verified here, neither for the nocturnal gregariousness, nor for the breeding sociality. However, it should be noted that most of the short-lived bird species of our dataset were songbirds, which defend a nesting territory during the breeding season (and thus do not show any gregariousness tendency during this period). Finally, no significant link between seropositivity risk and migratory status was observed, although it had been the case for two datasets we used [20,22]. However our analyses focused on short-lived bird species, where the proportion of birds borne during the year of sampling is high, therefore blood-sampled before their first migration to Africa. An additional possible explanation may be the inclusion of studies done during West Nile outbreaks or intense circulation periods, for example in Italy between 2006 and 2013 [57,58] when resident species may present very high prevalence of antibodies.

The traits model was used to predict seroprevalence in each of the 150 short-lived European bird species. Considering seroprevalence as a proxy for the species potential implication in WNV circulation, these predictions were then combined with maps of species distribution, to obtain a risk map at the continental level. This map combined risk level and confidence level, as it represented the geographical variations of the probability of high-risk areas across Europe, a high-risk area being defined as an area for which the risk is above the 90^th^ percentile of the risk distribution at the continental scale. Although only based on bird data (species-specific risk and species distribution), this map highlight several areas where WNV had been shown to circulate in the recent years, such as Morocco, Tunisia, Greece, Romania, Hungary, Italy, Southern France or Spain. The logistic model satisfactorily fitted the WND case occurrence dataset and showed a good ability to predict the occurrence of WND cases in districts of the European Union, as most of the districts having reported WND cases were correctly classified by the model. There are two main limitations to our study: (i) the detection sensitivity was assumed similar in all countries of concern but human cases may have been missed in countries classified at risk by the model ii) most of serosurveys included in the analyses were performed after WN human cases occurrence. The model also predicted a significant risk of WND cases in districts where no WND case had been reported, such as Poland. This was an expected result as the present study only investigated the reservoir host (i.e. bird-borne) component of WND structural risk. Some areas may present a high risk level for the bird-borne component if bird species assembly is favourable to WNV circulation, but a low global WND structural risk if the abundance of competent vectors is low, for example. Assessing the mosquito-borne structural risk and combine it with the bird-borne one is crucial will be crucial in the coming year to precisely identify areas at risk where vaccination and risk-based surveillance can be implemented. Some areas may also present a high structural but low conjectural risk. This may be the case in Poland, although some reports of WND cases in human in this country [59,60] suggest that WNV could have been occasionally circulating in specific areas. Furthermore, in this country, significant seroprevalence levels have recently been described in humans with meningitis and lymphocytic meningitis (14 of 42 cases), as well as in horses and wild birds [44]. Finally, as suggested by Zehender et al [61], WNV may need a few years of enzootic circulation before transmission to dead-end hosts: Poland may be in this situation. This time lag may be partially explained by the above–mentioned conjectural risk and justify a long term surveillance and awareness in risky areas.

Lastly, considering only VNT which is highly specific may partially explain why some currently affected areas were classified at low risk by the model.

To be more reliable, the present analysis need to be further completed with bird serological data collected without any human cases history the year before, weighted by a country-specific detection sensitivity and an analysis of the mosquito-borne structural risk.

## Conclusions

The study reported here allowed identifying several eco-ethological traits associated with WNV seroprevalence in European short-lived bird species, and using this statistical association and the bird species geographic distribution, to derive maps for the geographic variations of the bird-borne structural risk of WNV circulation in Europe. Comparison with case incidence data in human showed a strong association between bird-borne structural risk and occurrence of WND cases. Although the bird-borne structural risk, alone, is not sufficient to predict the occurrence of WND cases, it can be expected that, in the future, the combination of this risk with the vector-borne structural risk may allow identifying areas where the risk of WND occurrence is high.

## Supporting information

S1 Table Potential risk factors for WNV seropositivity in European bird species weighing <50g and predicted seroprevalence according to the traits-based model. (XLSX)

S2 Table Habitat preferences of European bird species weighing <50g. (XLSX)

S3 Table PRISMA checklist completed for the present analysis (from [28]). (DOC)

S4 Table WNV seroprevalence data in European bird species weighing <50g. (XLSX)
